# Serum concentrations of C-reactive protein (CRP) in lean and overweight dogs

**DOI:** 10.1186/1751-0147-57-S1-O15

**Published:** 2015-09-25

**Authors:** Anna Hillström, Josefin Söder, Sara Wernersson, Ragnvi Hagman

**Affiliations:** 1 Department of Clinical Sciences, Swedish University of Agricultural Sciences, Uppsala, Sweden; 2 Department of Anatomy, Physiology and Biochemistry, Swedish University of Agricultural Science, Uppsala, Sweden

## Introduction

Obesity in dogs has been associated with both increased and decreased blood C-reactive protein (CRP) concentrations.

## Objectives

To compare serum CRP concentrations between lean and overweight healthy dogs, and to investigate if results are affected by how dogs are classified as healthy; by owner interview only, or in combination with physical examination and laboratory testing.

## Methods

28 adult male intact Labrador Retrievers, reported to be healthy by their owners, were included. 12 dogs were categorized as lean (body condition score, BCS, ≤5) and 16 as overweight (BCS ≥6). Physical examination and laboratory testing were performed. Serum CRP concentrations were measured with a canine-specific high-sensitivity CRP assay (Gentian hsCRP). Mann Whitney U test (⍺=0.05) was used for data analyses, performed before and after exclusion of dogs with any abnormality detected.

## Results

The median (range) CRP concentration was 1.0 (<0.5-10.0) mg/l in lean and 1.1 (0.5-6.4) mg/l in overweight dogs (p=0.87). Minor abnormalities were found at physical examination in 11 dogs, and increased alanine aminotransferase (ALAT) values were detected in 2 dogs. After excluding these dogs, the median CRP concentrations in lean (n=4) and overweight (n=11) dogs were 0.6 (<0.5-1.4) mg/l and 1.1 (0.5-6.4) mg/l, respectively (p=0.17).

## Conclusions

CRP concentrations did not differ significantly between lean and overweight dogs. Physical examination revealed minor abnormalities in over 1/3 of the dogs, despite owners' assessment as healthy. To avoid abnormalities that could affect results when studying CRP in canine obesity, clinical assessment of health status is important.

